# Discovery and characterisation of a new leaf rust resistance gene introgressed in wheat from wild wheat *Aegilops peregrina*

**DOI:** 10.1038/s41598-020-64166-2

**Published:** 2020-05-05

**Authors:** Deepika Narang, Satinder Kaur, Burkhard Steuernagel, Sreya Ghosh, Urmil Bansal, Jianbo Li, Peng Zhang, Subhash Bhardwaj, Cristobal Uauy, Brande B. H. Wulff, Parveen Chhuneja

**Affiliations:** 1 School of Agricultural Biotechnology, Punjab Agricultural University, Ludhiana, Punjab 141004 India; 2 John Innes Centre, Norwich Research Park, Norwich, NR4 7UH UK; 3 The University of Sydney, Plant Breeding Institute Cobbitty, PMB4011, Narellan, NSW 2567 Australia; 4 School of Life Science and Technology, University of Electronic Science and Technology of China, Chengdu, 610054 Sichuan China; 5 ICAR – Indian Institute of Wheat and Barley Research, Regional Station, Flowerdale, Shimla, India

**Keywords:** Agricultural genetics, Plant molecular biology

## Abstract

Wild wheat species *Aegilops peregrina* (U^p^U^p^S^p^S^p^), harbours resistance to various diseases including leaf rust and stripe rust. Inheritance studies in a recombinant inbred line population of wheat*-Ae. peregrina* introgression line IL pau16061 revealed the transfer of a single major dominant gene conditioning all stage resistance, herein temporarily designated as *LrAp*. Genomic *in situ* hybridisation of IL pau16061, resistant and susceptible RILs with U- and S-genome DNA probes confirmed that the introgression with leaf rust resistance is from the U^p^ genome of *Ae. peregrina*. Fluorescence *in situ* hybridisation using chromosome specific probes identified U^p^ genome introgression to be on the long arm of wheat chromosome 6B. To genetically map *LrAp*, bulked segregant analysis was combined with resistance gene enrichment sequencing (MapRenSeq). Five nucleotide binding leucine-rich repeat contigs distinguished resistant and susceptible bulks and single nucleotide polymorphism (SNP) markers from these contigs co-segregated with *LrAp*. All five RenSeq NB_ARC contigs showed identity with the long arm of wheat chromosome 6B confirming the introgression on 6BL which we propose is a compensating translocation from *Ae. peregrina* chromosome 6U^p^L due to homoeology between the alien and wheat chromosomes. The SNP markers developed in this study will aid in cloning and marker assisted gene pyramiding of *LrAp*.

## Introduction

Wheat is the prime food commodity of major part of the world’s population with an annual harvest of 733 million tons in 2015. It is a leading source of calories as well as protein both for humans and livestock^1^. The limited genetic diversity at the farmers’ field increases genetic vulnerability to various biotic and abiotic stresses^2^. Almost wherever wheat is grown, production is significantly constrained by one or more of the three rust diseases^3^. These include leaf rust (*Puccinia triticina* Eriks); stem rust (*P. graminis* Pers. f. sp*. tritici*); and stripe rust (*P. striiformis* Westend f. sp. *tritici*). Among the three wheat rust pathogens, leaf rust cause less damage as compared to stripe and stem rust, however, due to its recurrent global occurrence, it would be anticipated that total annual losses incurred would be greater than any other rusts^4,5^. In India, stem rust and stripe rust are geographically constrained, while leaf rust is endemic across all production areas. The disease not only reduce the kernel weight but also lessen number of kernels per head^6,7^. The frequent emergence of novel pathotypes of *P. triticina* (Pt) and switching of virulence patterns constitute the main hurdles for its management^8,9^.

Wild species of wheat, especially members of the tertiary gene pool, carry an immense diversity of disease resistance (*R*) genes that could enable more sustainable disease control^10,11^. Till date, 79 genes for leaf rust resistance have been designated and ~44% are from wild progenitor and non-progenitor species^12^. These genes include *Lr21, Lr22a, Lr32, Lr39/Lr41, Lr40, Lr42, Lr43* (*Ae. tauschii*); *Lr28, Lr35, Lr36, Lr47, Lr51, Lr66* (*Ae. speltoides*); *Lr63* (*T. monococcum*); *Lr53, Lr64* (*T. dicoccoides*); *Lr18, Lr50* (*Triticum timopheevi*); *Lr37* (*Ae. ventricosa*); *Lr9, Lr76* (*Aegilops umbellulata*); *Lr19*, *Lr24*, *Lr29* (*Thinopyrum ponticum*); *Lr25, Lr26* (*Secale cereale*); *Lr38* (*Th. intermedium*); *Lr54* (*Ae. kotschyi*); *Lr55* (*Elymus trachycaulis*); *Lr56* (*Ae. sharonensis*); *Lr57* (*Ae. geniculata*); *Lr58* (*Ae. triuncialis*); *Lr59* (*Ae. peregrina*) and *Lr62* from *Ae. neglecta*^11,12^ besides a number of undesignated genes.

*R* genes from wild relatives are often linked to genes conferring undesirable traits. It typically takes many years to break this linkage drag and for *R* genes to be introduced into breeding programs and get deployed. Difficulties associated with genetic characterisation of alien genes include low throughput and physical resolution of cytogenetic techniques^10,13,14^ and limited transferability of microsatellite markers to the tertiary gene pool^15^. These factors limited the utilisation of these attractive resources for wheat improvement. However, the recent developments in genomic approaches and the accessibility of various genome sequences has allowed the fast access to genes in wild species.

Majority of the cloned *R* genes code for proteins with nucleotide-binding and leucine-rich repeat (NLR) domains^16–18^ that either in direct or indirect fashion recognise avirulence proteins. Resistance gene enrichment sequencing (RenSeq) of this particular gene class entails hooking of fragments from a genomic or cDNA library utilizing tailored biotinylated RNA oligonucleotides that complements the genes encoding for NLRs of a reference genome^19,20^. RenSeq on resistant and susceptible bulks developed from two populations of potato, each depicting segregation for a single *R* gene to *Phytophthora infestans*, led to the recognition of SNPs in NLR genes associated with resistance^19^. Converting RenSeq SNPs into Kompetitive Allele-Specific PCR (KASP) genotyping markers makes the RenSeq approach even more robust for mapping and tracking candidate *R* genes.

A leaf rust resistant introgression line (IL) pau16061, was developed previously from the hybridisation of the wild species *Ae. peregrina* (U^p^U^p^S^p^S^p^) with the wheat line WL711 (vulnerable to rust)^21^. In the present study, the wheat*-Ae. peregrina* IL pau16061 was characterised cytogenetically to identify the donor genome of the introgression with leaf rust resistance, and the resistance was mapped with SNP markers designed directly from NLRs identified through MapRenSeq.

## Results

### Inheritance studies

**Figure 1:**
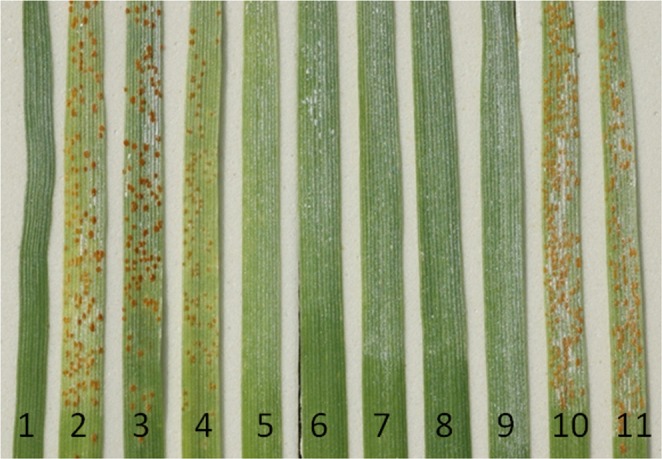
Leaf rust reaction at the seedling stage against leaf rust pathotype 77-5; 1 (*Ae. peregrina*); 2 (Agra Local); 3 (WL711); 4 (PBW343); 5 (IL pau16061); 6-9 (resistant progenies); 10-11(susceptible progenies). Seedling stage screening was conducted against *Pt* 77-5 in a glasshouse in PAU, Ludhiana, India. ITs ranging from ‘0’ to ‘2’ considered as resistant and ITs ranging from 3 to 3+ considered as susceptible^38^.

IL pau16061 was resistant to the majority of the leaf rust pathotypes prevalent in the Indian subcontinent *Pt*11, *Pt*12-5, *Pt*16-1, *Pt*77-5,* Pt*77-8, *Pt*77-10, *Pt*104-2,* Pt*104B, *Pt*106 and *Pt*162-2 at the seedling stage while WL711 was highly susceptible. The IL pau16061/WL711 F_5:6_ RIL population along with parental lines was tested at the seedling stage against *Pt* pathotype 77-5. The F_1_ plants obtained from this cross were resistant with near-immune infection type;, indicating the dominant behavior of the leaf rust resistance gene *LrAp*. Twelve seedlings from each of 160 F_5:6_ families were screened. All the seedlings in 69 families showed resistant infection type (;) and these RILs were classified as homozygous resistant, while all the seedlings in 81 families showed infection type 33+ and these RILs were classified as homozygous susceptible (Fig. 1)^22^. Ten families were found to to be segregating for the leaf rust resistance gene. Seedlings were transplanted in the field and tested for terminal disease severity. At adult plant stage, WL711 showed terminal disease severity of 80 S, whereas *Ae. peregrina* and IL pau16061 were highly resistant (0-TR). Out of the 160 RILs, 69 were totally free from leaf rust (0) whereas 81 showed leaf rust severity of 60S-80S, and 10 were found segregating. The segregation pattern confirmed a monogenic inheritance of leaf rust resistance (χ^2^_1.875:0.25:1.875_ = 0.96, *p* = 0.618) and the resistance locus was tentatively designated as *LrAp*.

### *In situ* hybridisation studies

**Figure 2:**
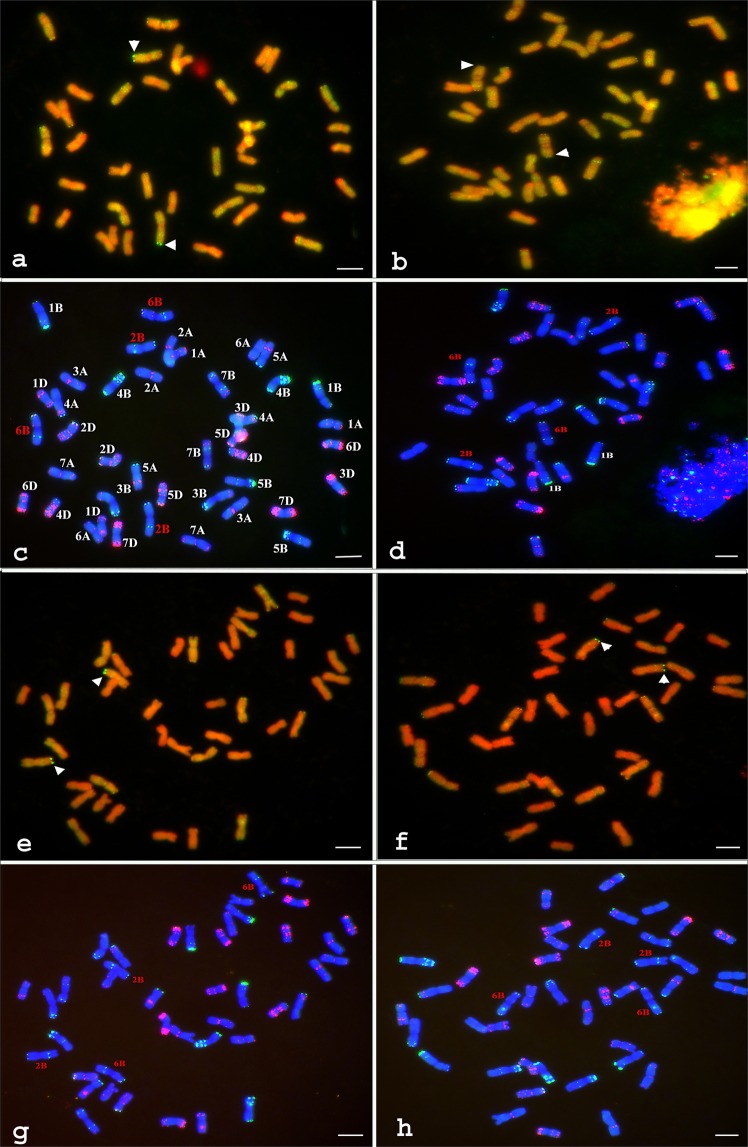
GISH and FISH on the metaphase cells of IL pau16061 (**a–d**), resistant (**e,g**) and susceptible RILs (**f**, **h**). For GISH, the DNA was labeled with Biotin-16-dUTP and signals were detected with fluorescein-avidin DN, which fluoresces yellow-green. Chromosomes were counterstained with 4′,6-diamidino-2-phenylindole (DAPI) and pseudocolored red. Arrowheads point to the hybridisation signals. For FISH, two probes labelled with FITC and Rhodamine were used to generate chromosome specific hybridisation signals. Chromosomes were counterstained with DAPI. (**a**) GISH on IL pau16061 using total genomic DNA from *Aegilops speltoides;* (**b**) GISH on IL pau16061 using total genomic DNA from *Ae. umbellulata;* (**c**,**d**) Sequential FISH of the same metaphase spreads using probes Oligo-pSc119.2-2 and Oligo-pTa535-2 labelled with 6-carboxyfluorescein (6-FAM) or 6-carboxytetramethylrhodamine (Tamra). Chromosomes have been identified from the banding pattern generated by the hybridization signals as reported by Tang *et al*.^47^; (**e**) GISH using S genome DNA as probe on a resistant RIL; (**f**) GISH using S genome DNA as probe on a susceptible RIL; (**g**) Sequential FISH using Oligo-pSc119.2-2 and Oligo-pTa535-2 probes on the same cell in the R RIL as in (**e**); (**h**) FISH using Oligo-pSc119.2-2 and Oligo-pTa535-2 probes on the same cell in the S RIL as in (**f**).

To identify the genomic origin of the *Ae. peregrina* introgression in IL pau16061, i.e. whether from the U^p^ or S^p^ genome, GISH was performed with genomic DNA from the U- and S-genome diploid species *Ae. umbellulata* and *Ae. speltoides*, respectively, as probes. Utilising S-genome DNA as probe, a strong signal was detected e distally in one pair of sub-metacentric chromosomes (Fig. 2a). GISH using U-genome DNA also detected a weak signal on the distal region of the long arm of a pair of chromosomes with satellites (Fig. 2b) indicating that IL pau16061 carries introgression from both the U^p^ and S^p^ genomes on two different pairs of chromosomes. To identify the chromosomes carrying the U and S genome introgressions, sequential FISH was conducted on the same metaphase spreads on which U and S genome introgressions were detected with GISH. S genome introgression was confirmed to be on the long arm of wheat chromosome 2B (Fig. 2c) while the U genome introgression was identified to be on the long arm of wheat chromosome 6B (Fig. 2d). GISH using S-genome probe was also conducted on 11 homozygous resistant (HR) and 11 homozygous susceptible (HS) RILs to identify whether the U or S genome introgression carries leaf rust resistance. Strong hybridisation signals were detected in both the HR (Fig. 2e) and HS lines (Fig. 2f), indicating that the S-genome introgression does not carry leaf rust resistance. Sequential FISH also confirmed the recombinant chromosome with GISH signals to be 2B in both HR and HS RILs (Fig. 2g,h). That the U genome introgression confers leaf rust resistance was further confirmed with FISH because a very strong hybridisation site with probe Oligo-pTa535-2 at the very distal end of 6BL was missing in IL pau16061 (Fig. 2c,d) and resistant RILs (Fig. 2g) but present in the susceptible RILs (Fig. 2h). Loss of a wheat specific signal at the telomere of 6BL indicated that the distal end of 6BL in IL pau16061 and resistant lines was replaced by a U-genome introgression. In conclusion, it is U-genome introgression in 6BL that confers the resistance to leaf rust in IL pau16061.

### NLR enrichment, sequencing and assembly

For mapping of *LrAp*, MapRenSeq was employed, the sequential strategy of which is outlined in Supplementary Fig. 1. We performed short read NLR enrichment on IL pau16061, the recurrent susceptible parent WL711, and F_5:6_ RB and SB. Quantitative PCR on the enriched libraries indicated a 500 to 1,000-fold increase in NLRs relative to other genes. The enriched libraries were paired-end sequenced on the Illumina HiSeq2500 platform. *De novo* assembly of short reads using CLC resulted in 3,586 NLR contigs (287 of which could be annotated as complete) after running through NLR–parser (Supplementary Table S1). Paired-end read (125 bp) data of susceptible parent and bulks ranged from 1.0 to 1.2 Gb for these libraries, which corresponded to an average NLR coverage of ~100×.

### Mapping *LrAp* with NLR markers

RenSeq BAM files of the resistant parent, susceptible parent and bulks were imported into the Savant genome browser and 3,586 NLR containing contigs were manually inspected to identify polymorphisms. We identified five contigs with SNPs with an allele frequency indicative of linkage to *LrAp*, i.e. a majority of SNPs derived from the susceptible parent were detected in the SB and a majority of SNPs derived from the resistant parent were detected in the RB (Supplementary Fig. 2). BLASTn searches of these five polymorphic RenSeq contigs against the IWGSC RefSeq v.1.0 assembly gave best hits with the two scaffolds of chromosome 6B. Out of five contigs, four contigs, namely ctg_1473_3, ctg_2659_1, ctg_1087_1 and ctg_765_1, showed a best BLAST hit on 6BL scaffold 119265 at positions 713.06 Mb, 713.14 Mb, 713.78 Mb and 713.96 Mb, respectively, while contig_5109_1 gave a best hit on scaffold 151695 at position 715.97 Mb (Supplementary Table S2).

To validate the putative SNPs and to map *LrAp* with these markers, we designed KASP assays for five SNPs in NLRs from these contigs. When these KASP markers were applied to the RIL population and the parents, they all formed distinct clusters, clearly separating IL pau16061 specific alleles present in one group and WL711 specific alleles in the other group. The primer sequences for KASP assays are listed in Supplementary Table S3.

**Figure 3:**
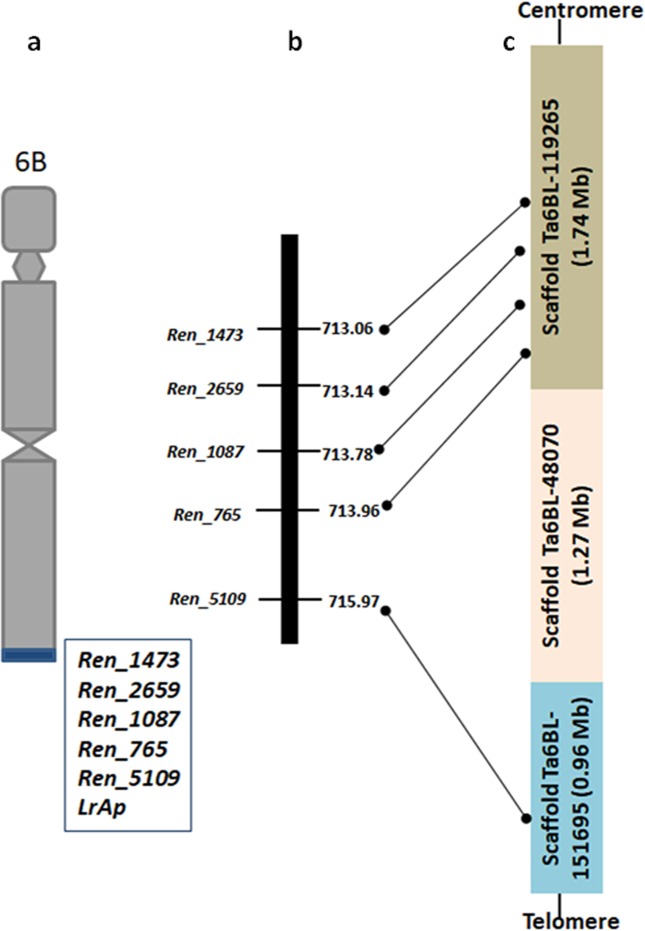
Mapping of *Ae. peregrina* leaf rust resistance gene *LrAp*. (**a**) Recombinant chromosome 6B of wheat-*Ae. peregrina* introgression line pau16061. *Ae. peregrina* introgression is depicted in blue at the distal end of 6BL. Co-segregating markers derived from NLR contigs along with leaf rust resistance gene *LrAp* are shown in the box. (**b**) Physical order of the RenSeq markers on telomeric region of 6BL according to wheat RefSeq v1.0 assembly; (**c**) candidate scaffolds of 6BL to which polymorphic NLR contigs were anchored from which RenSeq markers were developed.

The linkage analysis of the RenSeq markers and phenotypic data indicated that *LrAp* was cosegregating with these loci indicating the location of all five markers on the alien segment which did not recombine with its wheat counterpart (Fig. 3). Most proximal polymorphic marker Ren_1473 was physically mapped at 713.09 Mb and distal most marker at 715.97 Mb with reference to the wheat RefSeqv1.0 (IWGSC 2018) delineating alien segment to this 2.91 Mb region at the telomeric end of long arm of wheat chromosome 6B. Distal end of chromosome 6BL would be considered as a hotspot for disease resistance genes as 3.00 Mb region encompass 52 genes out of which 34 genes have a signature NB_ARC domain of resistance genes while comparing with high confidence gene annotations in wheat RefSeqv1.1.

## Discussion

In wheat breeding, the identification, characterisation, mapping and deployment of new major rust resistance genes remains a crucial activity in the constant battle against evolving pathogen populations. Previously, transfer of one stripe rust and two leaf rust resistance genes from *Ae. peregrina* accession pau3519 to cultivated wheat have been reported^21^. The present study describes the cytogenetic characterisation and genetic mapping of the leaf rust resistance gene in the wheat-*Ae. peregrina* introgression line IL pau16061 using exome capture of NLRs (MapRenSeq). Inheritance studies showed that the leaf rust resistance to pathotype 77-5 in IL pau16061 is conferred by a single major dominant gene (*LrAp*). *LrAp* mediated resistance is expressed at both the seedling and adult plant stages making it an all stage resistance (ASR) gene. Using GISH and FISH on IL pau16061, we identified that the leaf rust resistance is conferred by an introgression on the long arm of wheat chromosome 6B from the U^p^ genome of *Ae. peregrina*. Because the introgression lines were developed through induced homoeologous pairing^21^, most likely it is a compensating translocation from chromosome 6U^P^L.

Various studies have used high-throughput genotyping combined with bulked segregant analysis (BSA) to identify molecular markers associated with traits of interest^23^. For example, combined RNA-Seq with BSA to identify SNPs associated with the stripe rust resistance gene *Yr15* introgressed into wheat from *T. dicoccoides*^24^. However, RNA-Seq is biased by the tissue sampled, the time of sampling and limited by the sequencing depth. This problem is further confounded in the case of NLRs which are often clustered in complex arrays, display extreme sequence and copy number variation between different accessions due to diversifying selection^25^ and are expressed at relatively low levels^24,26^.

To mitigate these obstacles, we combined BSA mapping with NLR exome capture and sequencing (dubbed MapRenSeq) to define NLRs associated with *LrAp*. This strategy was previously used in the wild diploid potatoes *Solanum berthaultii* and *S. ruiz-ceballosii* to map *Rpi-ber2* and *Rpi-rzc1* conferring resistance to late blight^19^. By capturing and sequencing long NLR fragments using Pacific Biosciences SMRT technology^27^, improved the resolution of NLR assembly in MapRenSeq. This allowed the fine mapping and cloning of the late blight resistance gene *Rpi-amr3i* in the wild diploid potato *S. americanum*. In another variation of the technology, RenSeq was applied to multiple independently derived ethyl methanesulfonate (EMS) mutants of the stem rust resistance genes *Sr22* and *Sr45* introgressed into hexaploid wheat, which allowed the direct identification of single candidate genes without fine mapping^28^.

In the present study, we used MapRenSeq to characterise the alien segment carrying the *LrAp* gene conferring leaf rust resistance in wheat IL pau16061. The approach allowed the development of five co-segregating NLR_SNP markers to *LrAp*. The linked contigs could be positioned by sequence homology search onto two scaffolds, Ta6BL-151695 and Ta6BL-119265, on the long arm of chromosome 6B of the IWGSC RefSeq v.1.1 assembly. *LrAp* thus has been introgressed from the U^p^ genome of *Ae. peregrina* into the long arm of 6B. Both molecular cytogenetic studies and MapRenSeq based mapping unequivocally identified the introgression to be on the wheat chromosome arm 6BL. Previously, due to insufficient SSR marker density at terminal regions we were unable to identify any linked marker with *LrAp*^21^. However, MapRenSeq allowed direct access to NLR markers on the terminal alien segment. All the five NLR contigs polymorphic in RB and SB did not recombine with *LrAp* in 160 RILs indicating one of these might be carrying a putative candidate gene for *LrAp*. Delineating *LrAp* introgression will now allows this hypothesis to be tested by mutagenising IL pau16061 to obtain leaf rust susceptible mutants and sequencing these by MutRenSeq to clone *LrAp*^18,28,29^. Telomeric region of the wheat chromosome 6B was observed to be a hot spot of disease resistance genes as almost 52 disease resistance genes have been annotated in this region in wheat RefSeqv1.1 (https://urgi.versailles.inra.fr/).

To date, only two other leaf rust resistance genes have been transferred from *Ae. peregrina* to wheat, namely *Lr59* into chromosome arm 1AL^30^ and *LrP* linked with a stripe rust resistance gene, *YrP*, on wheat chromosome arm 5DS^31^ by our group. The original *Lr59*-containing translocation has group 6 chromosome homoeology at the distal end 6BS telomere (T1AS·1L^P^-6S^P^-6BS)^32^. The gene *LrAp* targeted in the present study is clearly different and new since no other published leaf rust resistance gene has been transferred from 6U^p^L of *Ae. peregrina* onto chromosome 6B of wheat. Only two *Lr* genes have been reported on the long arm of 6B viz. *Lr3* from *T. aestivum*^33^ and *Lr9* from the U genome of *Ae. umbellulata*^34,35^. The relationship, if any, between *LrAp* and *Lr9* will require further studies.

The identification of a co-segregating markers for *LrAp* in the present study will facilitate its marker-assisted selection and pyramiding with other major genes such as *Yr15, Yr5*, *Lr76/Yr70*, *Lr57/Yr40*, *Lr37/Yr17/Sr38, Yr36* and minor genes like *Lr34/Yr18/Sr57* to increase the potential for durable leaf rust resistance.

## Methods

### Plant materials

IL pau16061 was derived from the cross *T. aestivum* cv. Chinese Spring *Ph*^*I*^/*Ae. peregrina* pau3519//2*WL711(NN). Leaf and stripe rust susceptible recipient parent WL711 (NN), has the *kr* (crossability) alleles and is a near-isogenic line of *T. aestivum* cv. ‘WL711’. The strategy for developing introgression lines has been previously described in detail^21^. To study the inheritance pattern of rust resistance, IL pau16061 was crossed with WL711 (NN). The F_1_ was self-pollinated and a population of 160 F_5:6_ RILs was developed for genetic mapping of leaf rust resistance.

### Leaf rust screening

IL pau16061 and WL711 was screened against 10 *P. triticina* (*Pt*) pathotypes prevalent in the Indian subcontinent. Seedlings of RILs along with parental lines were screened against the *Pt*77-5^36^ in a glasshouse in the School of Agricultural Biotechnology, Punjab Agricultural University, Ludhiana, Punjab, India. The pathotype 77-5 is highly virulent, having overcome a number of leaf rust resistance genes in common wheat cultivars^37^. Seven-day-old seedlings were inoculated with urediniospores mixed with talc, incubated in humidified chambers for 16 h in the dark and then moved to a glasshouse maintained at 18-20 °C. Seedling reactions were recorded 14 days after inoculation and were classified according to the ‘0–4’ infection type (IT) scale^38^, with ITs ranging from ‘0’ to ‘2’ considered as resistant and ITs ranging from 3 to 3+ considered as susceptible. After recording the ITs, the seedlings were transplanted in the field in 1-meter rows to assess leaf rust severity at the adult plant stage. The susceptible cultivar WL711 was planted all around the experimental area. To maintain rust pressure, a mixture of urediniospores of leaf rust pathotypes 77-2, 77-5 and 104-2 was sprayed over the WL711 infector lines. Disease severity was recorded as the percentage of leaf area covered by rust following a modified Cobb’s scale^39^.

### Cytogenetic characterisation of IL pau16061 and homozygous resistant and homozygous susceptible lines

Genomic *in situ* hybridisation (GISH) was performed on IL pau16061 following the procedure of Zhang *et al*. (2001)^40^. Total genomic DNA from *Ae. umbellulata* (University of Sydney Cytogenetics collection C64.108) and *Ae. speltoides* (C64.69) were both labeled with Biotin-16-dUTP (Roche Diagnostic Australia, Castle Hill, NSW, Australia) using nick translation. Unlabeled total genomic DNA of wheat cv. Chinese Spring was used as a blocker. The probe to blocker ratio was ~1:80 and 1:120 for *Ae. umbellulata* and *Ae. speltoides*, respectively. Signals were detected with Fluorescein Avidin DN (Vector Laboratories, Burlingame, CA). Chromosomes were counterstained with 4′,6-diamidino-2-phenylindole (DAPI) (Invitrogen Life Science, Carlsbad, CA) and pseudo colored red. Slides were analyzed with a Zeiss Axio Imager epifluorescence microscope. Images were captured with a Retiga EXi CCD (charge-coupled device) camera (QImaging, Surrey, BC, Canada) operated with Image-Pro Plus version 7.0 software (Media Cybernetics Inc., Bethesda, MD) and processed with Photoshop version CS6 software (Adobe Systems, San Jose, CA).

Sequential Fluorescence *in situ* hybridisation (FISH) was performed on the same metaphase spreads using FITC- and Rhodamine- labelled probes, Oligo-pSc119.2-2 and Oligo-pTa535-2, respectively, for the identification of chromosomes according to Zhang *et al*. (2019)^41^.

### DNA extraction and quantification

Genomic DNA from young leaves of IL pau16061 (resistant parent), WL711 (susceptible parent) and the RILs was extracted using the CTAB method^42^ and quantified with Nanodrop and the Quant-iT PicoGreen dsDNA Assay kit (Life Technologies, Carlsbad, CA, USA). Two bulks were produced; resistant bulk (RB) and susceptible bulk (SB), comprising an equimolar concentration of DNA from the 20 most resistant RILs and the 20 most susceptible RILs, respectively.

### Illumina library preparation, enrichment and sequencing

A multiplexed NLR capture experiment was conducted on DNA of the resistant parent, susceptible parent, and RB and SB RILs. Short read libraries were prepared on these samples using the NEBNext Ultra DNA Library Prep Kit for Illumina (New England Biolabs, Inc., Ipswich, MA, USA) following the manufacturer’s instructions. Three to four samples were pooled prior to capture. Target NLR capture was carried out using a custom MYcroarray MYbaits kit v2.0 (MI, USA)^18^. Approximately 500 ng of the prepared libraries were hybridised in hybridisation buffer (10x SSPE, 10x Denhardt’s solution, 10 mM EDTA, 0.2% SDS) to the biotinylated RNA baits for 16-24 h at 65 °C. After hybridisation, bound DNA was recovered using magnetic streptavidin-coated beads and amplified through 8 cycles of PCR. Libraries were purified with AMPure XP beads and quantitative PCR was performed to establish the level of enrichment in NLRs relative to other non-target genes. The enriched libraries were paired-end sequenced on the Illumina HiSeq. 2500 platform at the Earlham Institute (previously The Genome Analysis Center, Norwich Research Park, UK). The raw data is available from NCBI, Study Numbers SRR8485686 (IL pau16061), SRR7867988 (WL711), SRR8369790 (resistant bulk) and SRR8382591 (susceptible bulk). The resistant parent was *de novo* assembled using CLC assembly cell (http://www.clcbio.com/products/clc-assembly-cell/) and default parameters. All contigs were run through NLR-parser^43^ to select those harboring NLR sequences.

### Genetic mapping of *LrAp* and chromosome assignment

Quality controlled reads of resistant parent, susceptible parent, RB and SB were mapped onto the assembled resistant parent contigs using Bowtie Wheeler’s Algorithm (BWA) with default settings^44^. Mapping data were visualised in the Savant Genome Browser (www.genomesavant.com). To identify linked polymorphisms, we looked into all NLR contigs for SNPs between the resistant and susceptible parents. Positive contigs with an alternate allele frequency of >70–80% in the susceptible parent and SB reads with an average coverage above 50 were identified. Regions with an average coverage less than 10% of the median overall coverage were considered as a presence/absence variation. Positive contigs were subsequently used in BLASTn searches (>80% identity) against the International Wheat Genome Sequencing Consortium (IWGSC) RefSeq v.1.0 (IWGSC 2018), to identify the most likely position in the wheat genome.

### Primer design

Primers were designed with Primer3 version 4.1.0^45^. The allele specific primers were designed in such a way that they encompass the parental polymorphism in the 3′ end. The reverse primers were selected 40-50 bp downstream specific to the required genome. For each SNP, two allele specific forward oligos with standard FAM or HEX compatible tails (FAM tail: 5′ GAAGGTGACCAAGTTCATGCT 3′; HEX tail: 5′ GAAGGTCGGAGTCAACGGATT 3′) and one common reverse primer were ordered. Primer mix was prepared by mixing 46 µL ddH_2_O, 30 µL common primer (100 µM) and 12 µL of each allele specific primer (100 µM)^46^. An assay of 5.07 µl volume included 2.5 µl of 2x KASP master mix (LGC, UK), 0.07 µl of primer mix and 30 ng of gDNA. PCR was performed as described by Ramirez *et al*. (2015)^24^. Optically clear plates (384-well) were read on a Tecan Safire plate reader. Fluorescence was observed at 36 °C for 30 s. For allelic discrimination KlusterCaller software v3.4.1.36 (LGC, UK) was used and three genotyping groups, FAM (homozygote), HEX (homozygote) and mixed (heterozygote), were observed.

### Statistical analysis and map construction

Chi-squared tests were performed to establish goodness of fit of observed segregation with the expected genetic ratio in the F_5:6_ RIL population and to detect marker-trait linkages. The positioning of markers was done manually based on recombination between genotypic and phenotypic data and on the basis of recombination frequency. Genetic distances were computed in centiMorgan (cM).

## Supplementary information

Supplementary Information.

## Data Availability

The datasets generated during and/or analysed during the current study are available at the NCBI, as under SRR8485686 (IL pau16061), [https://www.ncbi.nlm.nih.gov/sra/?term=SRR8485686], SRR7867988 (WL711), [https://www.ncbi.nlm.nih.gov/sra/?term=SRR7867988] SRR8369790 (resistant bulk), [https://www.ncbi.nlm.nih.gov/sra/?term=SRR8369790] and SRR8382591 (susceptible bulk), [https://www.ncbi.nlm.nih.gov/sra/?term=SRR8382591].

## References

[1] Mondal S (2016). Harnessing diversity in wheat to enhance grain yield, climate resilience, disease and insect pest resistance and nutrition through conventional and modern breeding approaches. Front Plant Sci..

[2] Wang C, Hu S, Gardner C, Lübberstedt T (2017). Emerging Avenues for Utilization of Exotic Germplasm. Trends Plant Sci..

[3] Hovmøller MS, Walter S, Justesen AF (2010). Escalating threat of wheat rusts. Science..

[4] Huerta-Espino J (2011). Global status of wheat leaf rust caused by *Puccinia triticina*. Euphytica..

[5] Savary S (2019). The global burden of pathogens and pests on major food crops. Nat Ecol Evol..

[6] Germán S (2007). The situation of common wheat rusts in the Southern Cone of America and perspectives for control. Aust J Agri Res..

[7] Herrera-Foessel SA (2011). New slow rusting leaf rust and stripe rust resistance genes *Lr67* and *Yr46* are pleiotropic or closely linked. Theor Appl Genet..

[8] Bhardwaj SC, Gangwar OP, Pramod P, Khan HICAR (2015). Indian Inst. of Wheat and Barley Research, Regional Station, Flowerdale, Shimla, India. Mehtaensis newsletter.

[9] Kiran K (2016). Draft genome of the wheat rust pathogen (*Puccinia triticina*) unravels genome-wide structural variations during evolution. Genome Biol Evol..

[10] Friebe B, Jiang J, Raupp WJ, McIntosh RA, Gill BS (1996). Characterization of wheat-alien translocations conferring resistance to diseases and pests: current status. Euphytica..

[11] Chhuneja, P. *et al* Introgression and exploitation of biotic stress tolerance from related wild species in wheat cultivars. In: Molecular Breeding for Sustainable Crop Improvement, Sustainable Development and Biodiversity, 11th edn. Sustainable Development and Biodiversity, Springer International Publishing, Switzerland (eds. Rajpal V. R. *et al*.) 269–324 (2016).

[12] McIntosh, R. A. *et al* Catalogue of gene symbols for wheat. In: KOMUGI - Wheat Genetic Resources Database at, https://www.shigen.nig.ac.jp/wheat/komugi/genes/download.jsp (2017).

[13] Friebe B (1991). Identification of alien chromatin specifying resistance to wheat streak mosaic virus and greenbug in wheat germplasm by C-banding and *in situ* hybridization. Theor Appl Genet..

[14] Lukaszewski AJ, Lapinski B, Rybka K (2005). Limitations of *in situ* hybridization with total genomic DNA in routine screening for alien introgressions in wheat. Cytogenet Genome Res..

[15] Mullan DJ (2006). EST-derived SSR markers from defined regions of the wheat genome to identify *Lophopyrum elongatum* specific loci. Genome.

[16] Dangl JL, Horvath DM, Staskawicz BJ (2013). Pivoting the plant immune system from dissection to deployment. Science.

[17] Kourelis J, Van der Hoorn RAL (2018). Defended to the nines: 25 years of resistance gene cloning identifies nine mechanisms for R protein function. Plant Cell..

[18] Marchal C (2018). BED-domain containing immune receptors confer diverse resistance spectra to yellow rust. Nat Plants..

[19] Jupe F (2013). Resistance gene enrichment sequencing (RenSeq) enables reannotation of the NB-LRR gene family from sequenced plant genomes and rapid mapping of resistance loci in segregating populations. Plant J..

[20] Andolfo G (2014). Defining the full tomato NB-LRR resistance gene repertoire using genomic and cDNA RenSeq. BMC Plant Biol..

[21] Narang D, Kaur S, Saini J, Chhuneja P (2018). Development and molecular characterization of wheat-*Aegilops peregrina* introgression lines with resistance to leaf rust and stripe rust. J Crop Imp..

[22] Narang, D. Fine Mapping and identification of candidate gene(s) for leaf rust and stripe rust resistance introgressed in *Triticum aestivum* L. from *Aegilops peregrina*. PhD thesis, Punjab Agricultural University, Ludhiana, Punjab, India (2017).

[23] Michelmore RW, Paran I, Kesseli RV (1991). Identification of markers linked to disease-resistance genes by bulked segregant analysis: a rapid method to detect markers in specific genomic regions by using segregating populations. Proc. Natl. Acad. Sci. USA.

[24] Ramírez-González RH (2015). RNA-Seq bulked segregant analysis enables the identification of high-resolution genetic markers for breeding in hexaploid wheat. Plant Biotechnol J..

[25] Meyers BC, Kozik A, Griego A, Kuang H, Michelmore RW (2003). Genome-wide analysis of NBS-LRR-encoding genes in Arabidopsis. Plant Cell..

[26] Steuernagel, B. *et al*. Physical and transcriptional organisation of the bread wheat intracellular immune receptor repertoire. Preprint at https://www.biorxiv.org/content/10.1101/339424v1 (2018).

[27] Witek K (2016). Accelerated cloning of a potato late blight–resistance gene using RenSeq and SMRT sequencing. Nat Biotechnol..

[28] Steuernagel B (2016). Rapid cloning of disease resistance genes in plants using mutagenesis and sequence capture. Nat Biotechnol..

[29] Xing L (2018). Pm21 from *Haynaldia villosa* encodes a CC-NBS-LRR protein conferring powdery mildew resistance in wheat. Mol Plant..

[30] Marais GF, McCallum B, Marais AS (2008). Wheat leaf rust resistance gene *Lr59* derived from *Aegilops peregrina*. Plant Breed..

[31] Narang, D. *et al*. Fine mapping of *Aegilops peregrina* cosegregating leaf and stripe rust resistance genes to distal-most end of 5DS. *Theor Appl Genet*. 10.1007/s00122-019-03293-5 (2019). 10.1007/s00122-019-03293-5 30706082

[32] Pirseyedi SM (2015). Characterization of recombinants of the *Aegilops peregrina*-derived *Lr5*9 translocation of common wheat. Theor Appl Genet.

[33] McIntosh, R. A. *et al*. Catalogue of gene symbols for wheat. Proceedings of the 10th International Wheat Genetics Symposium, Paestum, Italy (2003)

[34] Sears E. R. The transfer of leaf rust resistance from Aegilops umbellulata to wheat. Brookhaven Symp Biol. No. 9, Genetics in Plant Breeding, pp 1–22 (1956).

[35] Sears ER (1961). Identification of the wheat chromosome carrying leaf rust resistance from *Aegilops umbellulata*. Wheat Inf Serv..

[36] Bansal M (2017). Mapping of *Aegilops umbellulata* - derived leaf rust and stripe loci in wheat. Plant Pathol..

[37] Saini RG, Kaur L, Kaur M (1998). Adult plant leaf rust *Puccinia recondita* resistance of known *Lr* genes against three virulence variants of race 77 from Indian subcontinent. Indian J Agri Sci.

[38] McIntosh, R. A., Wellings, C. R. & Park, R. F. Wheat Rusts: an Atlas of Resistance Genes. CSIRO publications, East Melbourne, Australia (1995).

[39] Peterson RF, Campbell AB, Hannah AE (1948). A diagnostic scale for estimating rust severity on leaves and stem of cereals. Can J Res Sect C Bot Sci..

[40] Zhang P, Friebe B, Lukaszewski AJ, Gill BS (2001). The centromere structure in Robertsonian wheat–rye translocation chromosomes indicates that centric breakage-fusion can occur at different positions within the primary constriction. Chromosoma.

[41] Zhang J (2019). A strategy for identifying markers linked with stem rust resistance in wheat harbouring an alien chromosome introgression from a non-sequenced genome. Theor Appl Genet.

[42] Saghai-Maroof MA, Biyashev RM, Yang GP, Zhang Q, Allard RW (1994). Extraordinarily polymorphic microsatellite DNA in barley: species diversity, chromosomal locations and population dynamics. Proc Natl Acad Sci USA.

[43] Steuernagel B, Jupe F, Witek K, Jones JDG, Wulff BBH (2015). NLR-parser: rapid annotation of plant NLR complements. Bioinformatics..

[44] Li H, Durban R (2009). Fast and accurate short read alignment with Burrows-Wheeler transform. Bioinformatics.

[45] Untergasser A (2012). Primer3–new capabilities and interfaces. Nucleic Acids Res..

[46] LGC Genomics. http://www.lgcgroup.com/services/genotyping (2013).

[47] Tang Z, Yang Z, Fu S (2014). Oligonucleotides replacing the roles of repetitive sequences pAs1, pSc119.2, pTa-535, pTa71, CCS1, and pAWRC.1 for FISH analysis. J Appl Genet.

