# Supplementary material for: Discovery and characterisation of a new leaf rust resistance gene introgressed in wheat from wild wheat Aegilops peregrina
Source: Sci Rep. 2020 May 5;10:7573. doi: 10.1038/s41598-020-64166-2 (PMC7200655; doi:10.1038/s41598-020-64166-2)
Supplement: Supplementary file 1 — Supplementary Information. [file 41598_2020_64166_MOESM1_ESM.docx]

**Discovery and characterisation of a new leaf rust resistance gene introgressed in wheat from wild wheat *Aegilops peregrina***

Deepika Narang^1^, Satinder Kaur^1^, Burkhard Steuernagel^2^, Sreya Ghosh^2^, Urmil Bansal^3^, Jianbo Li^3,4^, Peng Zhang^3^, Subhash Bhardwaj^5^, Cristobal Uauy^2^, Brande B. H. Wulff^2^ and Parveen Chhuneja^1#^

^1^ School of Agricultural Biotechnology, Punjab Agricultural University, Ludhiana, Punjab, 141004, India

^2^ John Innes Centre, Norwich Research Park, Norwich, NR4 7UH, UK

^3^ The University of Sydney Plant Breeding Institute Cobbitty, PMB4011, Narellan, NSW 2567, Australia

^4^ School of Life Science and Technology, University of Electronic Science and Technology of China, Chengdu 610054, Sichuan, China

^5^ ICAR – Indian Institute of Wheat and Barley Research, Regional Station, Flowerdale, Shimla, India

# corresponding author email: [pchhuneja@pau.edu](mailto:pchhuneja@pau.edu)

**Supplementary Table 1** Assembly statistics of wheat- *Aegilops peregrina* IL pau16061.

| Insert size | 544 bp | | |
| --- | --- | --- | --- |
| Sequencing chemistry | HiSeq 2500 (125 bp PE) | | |
| Number of reads | 53,288,986 | | |
| Sequence output | 2.89 Gb | | |
| On target | 1.1 Gb | | |
| N50 | 2,250 bp | | |
| Number of contigs | 3,586^*^/163,500 | | |
| **NLR complement** |  |  |  |
| Class | Complete | Partial | Total |
| CNL with N-terminal CC domain | 287 | 3,299 | 3,586 |

|  |
| --- |
|  |

**Supplementary Table 2** Polymorphic SNPs and their BLAST hit on chromosome 6B of the IWGSC RefSeq v.1.0 assembly.

| Contig name | BLAST_hit | Identity (Query length) | E-value | Genetic bin | Physical position (Mb) | |
| --- | --- | --- | --- | --- | --- | --- |
| Contig_2659_1 | Scaffold Ta6BL-119265 | 4783/4783 (4783) | 0 | 95.11 Cm | 713,149,894 – 713,145,112 |  |
| Contig_765_1 | Scaffold Ta6BL-119265 | 1664/1665 (1665) | 0 | 95.11 cM | 713,960,809 – 713,962,473 | |
| Contig_1087_1 | Scaffold Ta6BL-119265 | 1540/1540 (1540) | 0 | 95.11 cM | 713,788,714 – 713,787,175 | |
| Contig_1473_3 | Scaffold Ta6BL-119265 | 1095/1095 (1095) | 0 | 95.11 cM | 713,067,721 – 713,066,627 | |
| Contig_5109_1 | Scaffold Ta6BL-151695 | 3055/3055 (3055) | 0 | 96.77 cM | 715,975,599 –  715,978,653 | |

**Supplementary Table 3** KASP markers developed from candidate NLR contigs used for fine mapping *LrAp*

| Contig name^a^ | Marker expression | Primer name | Sequence (5'-3') | Allele | Parent |
| --- | --- | --- | --- | --- | --- |
| Contig_2659_1  (Ta6BL-119265) | Codominant | Ren_2659_A1 | CATGATTTTGCAAGGCTAGC | C | IL pau16061 |
|  |  | Ren_2659_A2 | CATGATTTTGCAAGGCTAGT | T | WL711 |
|  |  | Ren_2659_R | TGGTGTACGGTTGGCAATAT |  |  |
| Contig_5109_1  (Ta6BL-151695) | Codominant | Ren_5109_A1 | TGGGAATATACAACCCCATATCA | A | IL pau16061 |
|  |  | Ren_5109_A2 | TGGGAATATACAACCCCATATCG | G | WL711 |
|  |  | Ren_5109_R | TCCACTTCTCCGCCGCTT |  |  |
| Contig_765_1  (Ta6BL-119265) | Codominant | Ren_765_A1 | CCTTGAGCACATTGGAAATCATATC | C | IL pau16061 |
|  |  | Ren_765_A2 | CCTTGAGCACATTGGAAATCATATT | T | WL711 |
|  |  | Ren_765_R | AAGATGCCTTCACAACGTCT |  |  |
| Contig_1087_1  (Ta6BL-119265) | Codominant | Ren_1087_A1 | ATGGGTCAGGGTGATGCG | G | IL pau16061 |
|  |  | Ren_1087_A2 | ATGGGTCAGGGTGATGCA | A | WL711 |
|  |  | Ren_1087_R | AGCAGAACACCAACTCCTACT |  |  |
| Contig_1473_3  (Ta6BL-119265) | Codominant | Ren_1473_A1 | CAGATTTGGAAACTCAATTTGACCTC | C | IL pau16061 |
|  |  | Ren_1473_A2 | CAGATTTGGAAACTCAATTTGACCTA | A | WL711 |
|  |  | Ren_1473_R | ACTCTCTTTGATTTAACATGATCCTTC |  |  |

| ^a^SNP markers are named as: starting with Ren (Resistance gene enrichment) followed with contig’s name. |
| --- |
| F1 primer tail sequence for HEX allele: GAAGGTCGGAGTCAACGGATT |
| F2 primer tail sequence for FAM allele: GAAGGTGACCAAGTTCATGCT |

- ***De novo* assembly of introgression line**
- **Map reads to assembly**
- **Call variants**
- **Annotate contigs**


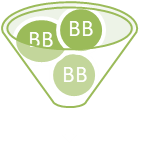


**Introgression line**

**Susceptible parent**

**Bulk resistant**

**Bulk susceptible**

- **Design and validate NLR markers**

**Enriched DNA**

**Total gDNA**

- **High throughput sequencing**

**Phenotyping**

**Bioinformatics**

**RenSeq**


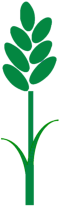

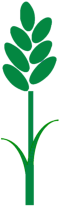

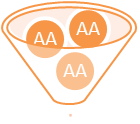


**Supplementary Fig. 1** Sequential outline of the MapRenSeq strategy to identify NLRs linked to *LrAp*. **a** Phenotyping and bulked genomic DNA preparations from homozygous resistant (BB) and homozygous susceptible (AA) RIL individuals determined on the basis of phenotypic data. **b** RenSeq using the NLR capture solution on the DNA from the bulks and parental lines. **c** Illumina sequencing of NLR-enriched DNA of the resistant parent (IL pau16061), the susceptible parent (WL711) and bulks, followed by bioinformatics identification of candidate NLRs linked to *LrAp* based on SNP frequency in RenSeq reads mapped against a *de novo* assembly of NLR sequences from IL pau16061. Candidate SNPs in NLRs linked to *LrAp* were verified by conversion to KASP markers and genotyping on the RIL population. ^22^


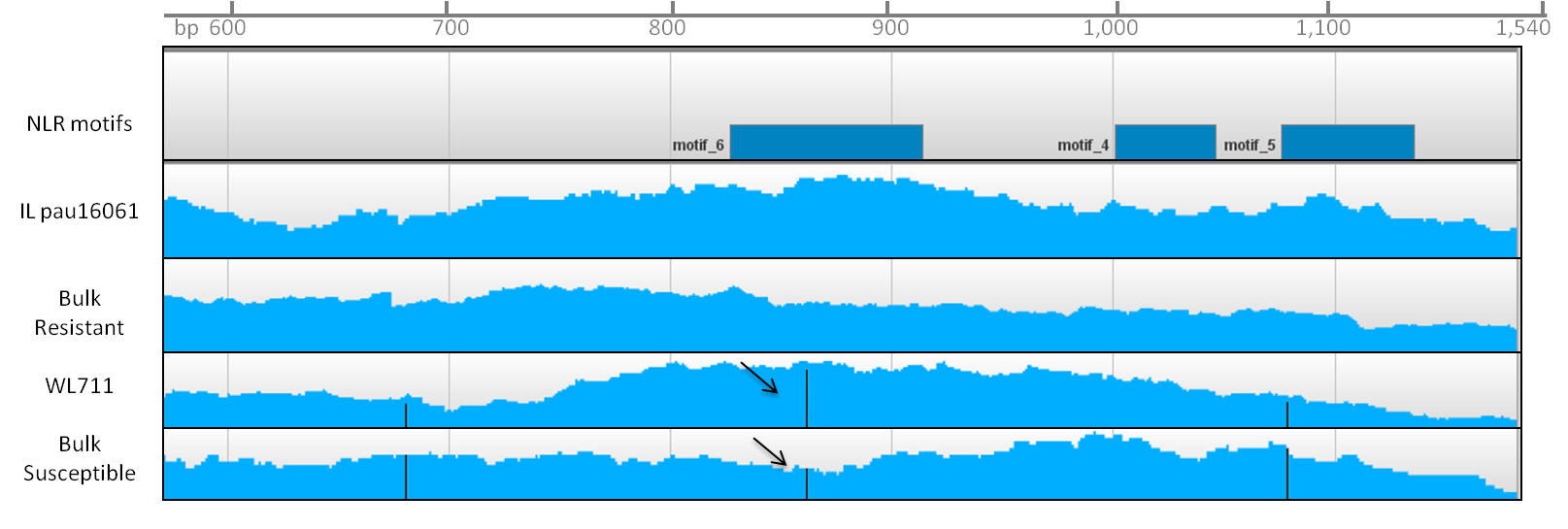


**Supplementary Fig. 2.** Visualisation of mapped RenSeq reads in Savant. Contig_1087_1 depicting NLR specific SNPs (marked in black). Blue background shows coverage. ^22^
